# Supplementary material for: Enhancing Proteoform Sequence Coverage Using Top-Down Mass Spectrometry with In-Source Fragmentation and Middle-Down Mass Spectrometry
Source: Anal Chem. 2026 Feb 2;98(5):3860–70. doi: 10.1021/acs.analchem.5c06097 (PMC12903053; doi:10.1021/acs.analchem.5c06097)
Supplement: Supplementary file 1 [file ac5c06097_si_001.pdf]

Enhancing proteoform sequence coverage using top-down mass spectrometry with in-source fragmentation and middle-down mass spectrometry (Supplemental Materials)

Xingzhao Xiong[1], Letu Qingge[2], Binhai Zhu[3] and Xiaowen Liu[1,*]

[1]Deming Department of Medicine, School of Medicine, Tulane University, New Orleans, Louisiana 70112, United States,

[2]Department of Computer Science, North Carolina A&T State University, Greensboro, North Carolina 27411, United States,

[3]Gianforte School of Computing, Montana State University, Bozeman, Montana 59717, United States.

*Corresponding author

Table of Contents

Supplemental Tables S2

Table S1. Parameter settings for TopFD S2

Table S2. Parameter settings for TopPIC S3

Table S3. Parameter settings for MSFragger S4

Supplemental Figures S5

Figure S1. Representative PrSMs for proteoforms with water loss S5

Figure S2. Representative MS1 spectra and XICs of the reference and ISF proteoforms of myoglobin under ISF energies 0V and 90V S6

Figure S3. Representative MS1 spectra and XICs of the reference and ISF proteoforms of CA2 under ISF energies 0V and 50V S7

Figure S4. Comparison the XICs of the reference proteoform and alternative reference proteoform S8

Figure S5. ISF proteoforms identified at different ISF voltage settings S8

Figure S6. Comparison of proteoform sequence coverage using representative PrSMs of single charge states versus combining representative PrSMs of multiple charge states S9

Figure S7. Comparison of proteoform sequence coverage from b-ions and y-ions across different ISF voltage settings S9

Figure S8. Percentages of b- and y-ions in proteoforms identified by top-down MS with various ISF energy settings S9

Figure S9. Distributions of the cleavage sites of fragment ions of proteoforms identified by top-down MS with various ISF energy settings S10

Figure S10. Fragment ion sequence coverage obtained using proteoforms identified by top-down MS with various ISF energy settings S11

Figure S11. Comparison of sequence coverage for myoglobin and CA2 in middle-down MS using five enzymes S12

Figure S12. Peptides and proteoforms of CA2 identified using MSFragger and TopPIC by middle-down MS with AspN digestion S12

Figure S13. Peptides and proteoforms of CA2 identified using MSFragger and TopPIC by middle-down MS with chymotrypsin digestion S13

Figure S14. Peptides and proteoforms of CA2 identified using MSFragger and TopPIC by middle-down MS with GluC digestion S14

Figure S15. Peptides and proteoforms of CA2 identified using MSFragger and TopPIC by middle-down MS with LysC digestion S14

Figure S16. Peptides and proteoforms of CA2 identified using MSFragger and TopPIC by middle-down MS with trypsin digestion S15

Supplemental Tables

Table S1: Parameters settings for TopFD

**Input Parameter**	**Value**
Version	1.7.9
Maximum charge	30 for ubiquitin and myoglobin, 40 for CA2
Maximum mass	40,000 Da
MS1 signal noise ratio	3.0
MS/MS signal noise ratio	1.0
M/*z* error tolerance	0.02
Min scan number in features	3
Use single scan noise level during feature extraction	True
ECScore cutoff	0.5
Fragmentation method	File
Disable additional feature search	True

Table S2: Parameters settings for TopPIC

TopPIC Parameter	Value
Version	1.7.9
**Proteome database**	Single protein sequence:<br>UniProt ID P0CH28 for ubiquitin<br>UniProt ID P68082 for myoglobin<br>UniProt ID P00921 for CA2
**Search type**	Target
**N-terminal forms of proteins**	NONE, NME, M_ACETYLATION, NME_ACETYLATION
**Use TopFD features**	Yes
**Fixed modifications**	Top-down: None;<br>Middle-down: Carbamidomethylation on cysteine
**Variable PTM**	Top-down: Water loss, -18.01 Da, all the 20 amino acids, anywhere<br><br>Middle-down: None
**Maximum number of variable modifications**	1
**Spectrum level cutoff type for filtering PrSMs**	E-value
**The cutoff value for filtering PrSMs**	0.01
**Spectrum level cutoff type for filtering proteoforms**	E-value
**The cutoff value for filtering proteoforms**	0.01
**Error tolerance for precursor and fragment masses**	10 ppm
**Error tolerance for identifying PrSM clusters**	1.2 Da
**Maximum number of unexpected mass shifts**	0
**E-values computation**	Generating function

Table S3: Parameters settings for MSFragger

**Input Parameter**	**Value**
Version	23.1
Precursor mass tolerance (ppm)	10
Fragment mass tolerence (ppm)	10
Mass calibration	Enable
Isotope error	0/1/2/3
Cleavage	Selected based the enzyme used in the experiments: AspN, chymotrypsin, GluC, LysC, or trypsin
Missed cleavage number	Myoglobin AspN: 10
	Myoglobin chymotrypsin: 30
	Myoglobin GluC, LysC, trypsin: 20
	CA2 AspN, LysC, trypsin: 20
	CA2 chymotrypsin: 50
	CA2 GluC: 30
Peptide length (amino acids)	6-100
Peptide mass (Da)	500-12000
Variable PTMs	N-terminal acetylation
Fixed modification	Cysteine carbamidomethylation
Maximum variable modification on a peptide	1
Minimum peaks	15
Use top N peaks	150
Minimum ratio	0.01
Intensity transform	None
Remove precursor peak	Only peak with precursor charge
Report mass shift as a variable modification	No
Minimum matched fragments	4
Deisotope	Yes
Deneutralloss	Yes

Supplemental Figures

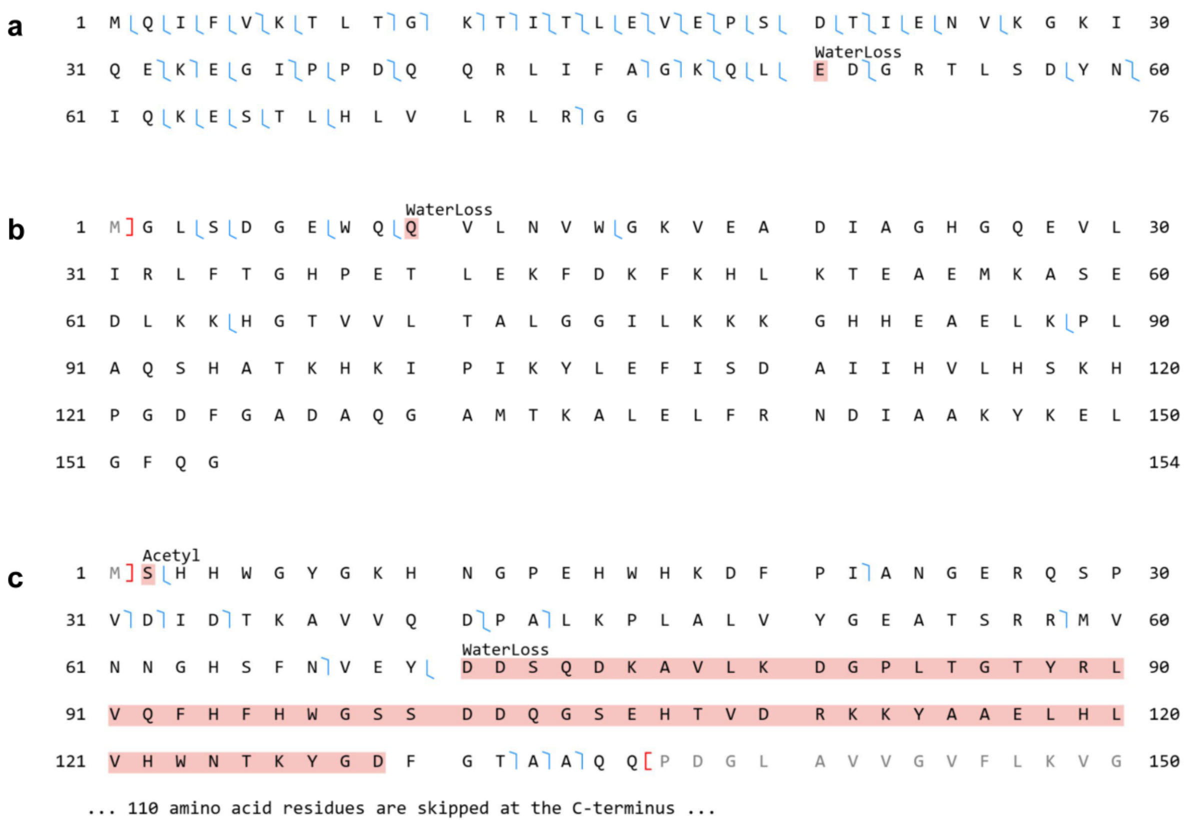

Fig. S1: Representative PrSMs for proteoforms with water loss. (a) Ubiquitin (residues 2–76), (b) myoglobin (residues 2–154), and (c) CA2 (residues 2–136).

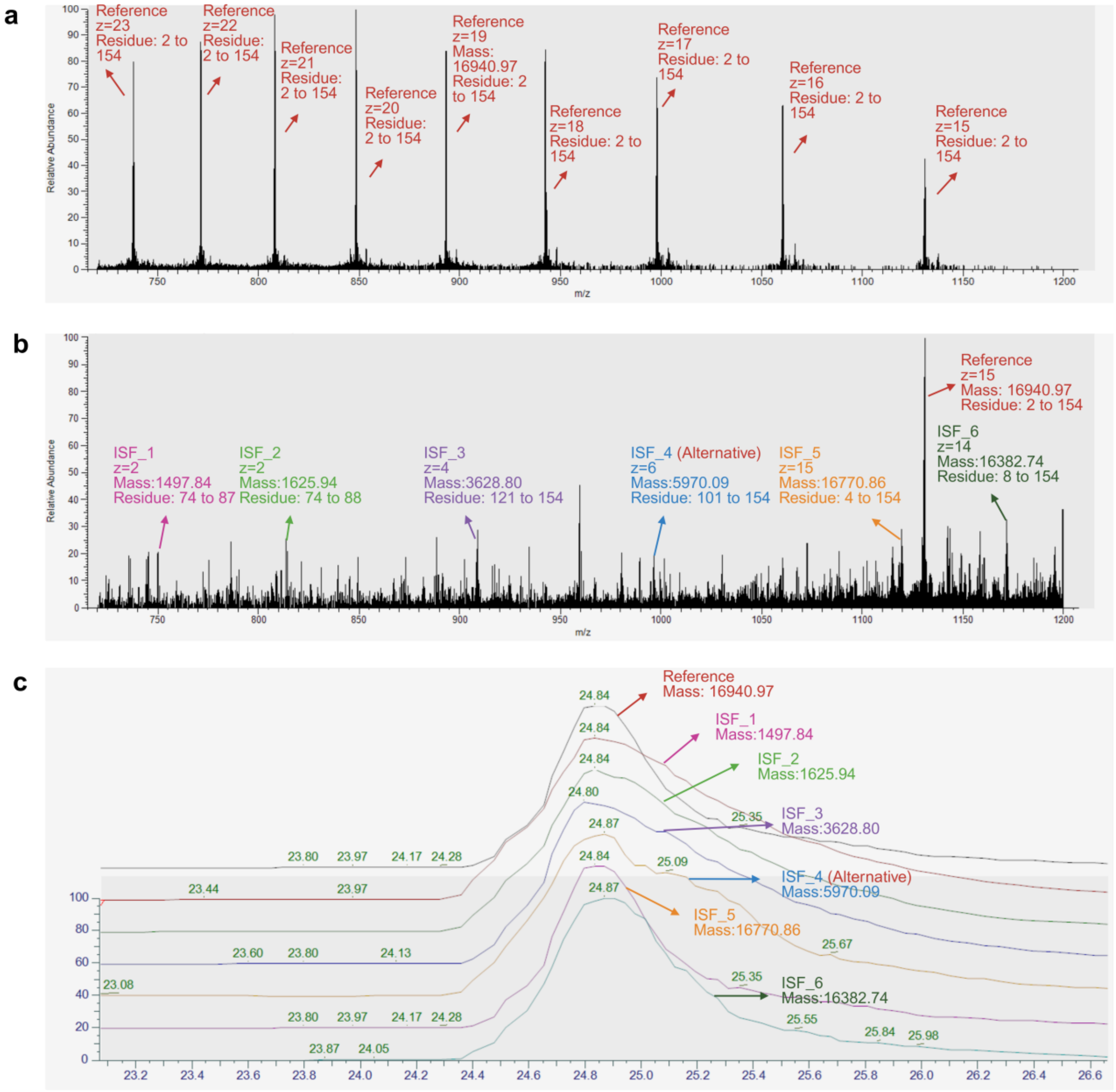

Fig. S2: Representative MS1 spectra and XICs of the reference and ISF proteoforms of myoglobin under ISF energies 0V and 90V. (a) Representative MS1 spectrum of the reference proteoform (residue 2-154) observed in an LC-MS run with 0V ISF energy. (b) Representative MS1 spectrum and (c) XICs of the reference proteoform and 6 ISF proteoforms observed in an LC-MS run with 90V ISF energy.

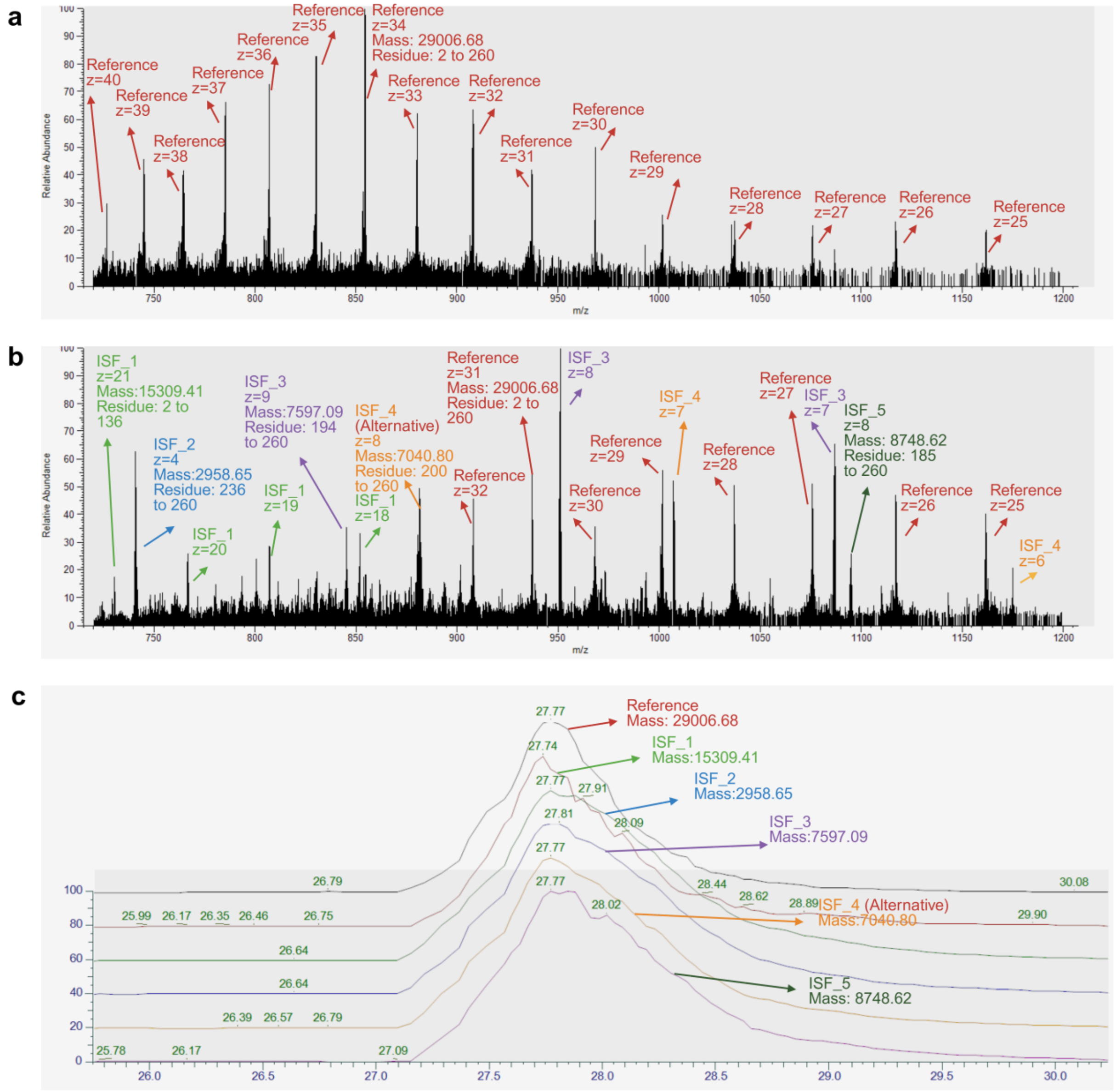

Fig. S3: Representative MS1 spectra and XICs of the reference and ISF proteoforms of CA2 under ISF energies 0V and 50V. (a) Representative MS1 spectrum of the reference proteoform (residue 2-260) observed in an LC-MS run with 0V ISF energy. (b) Representative MS1 spectrum and (c) XICs of the reference proteoform and 5 ISF proteoforms observed in an LC-MS run with 50V ISF energy.

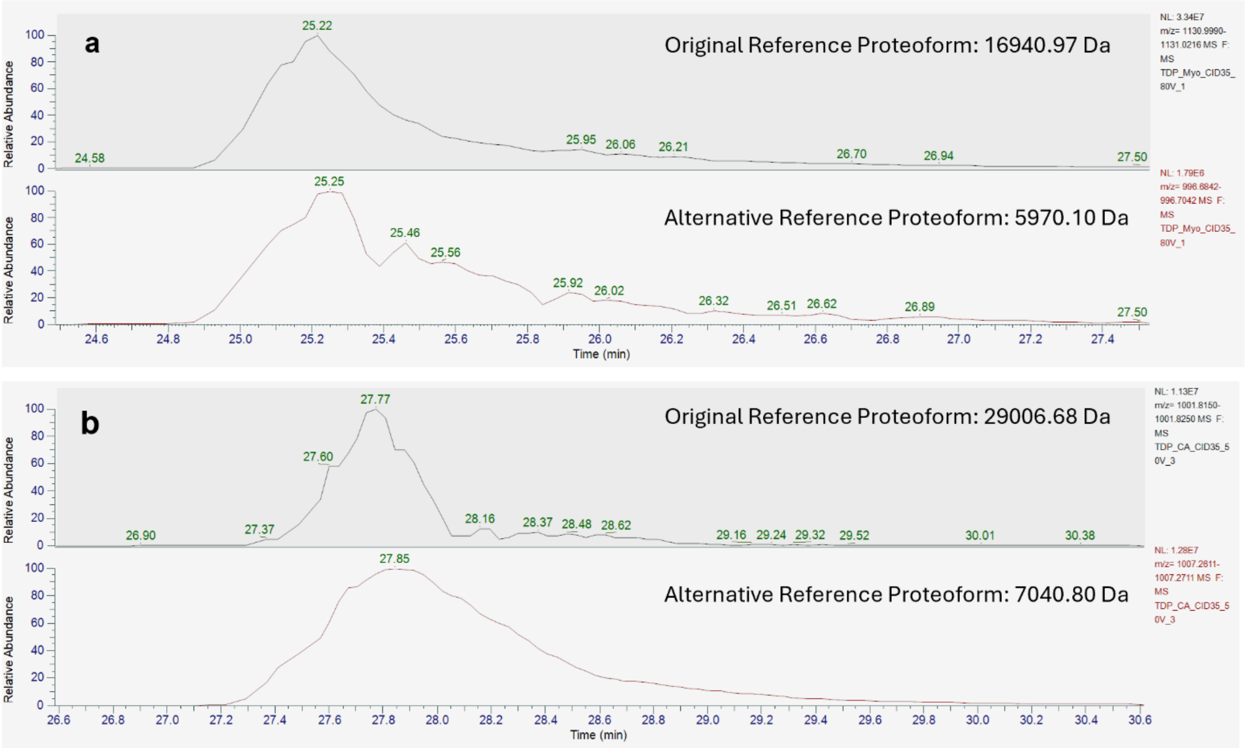

Fig. S4: Comparison the XICs of the reference proteoform and alternative reference proteoform. (a) An MS data file of myoglobin with an ISF voltage of 80 V and (b) an MS file of CA2 with an ISF voltage of 50 V.

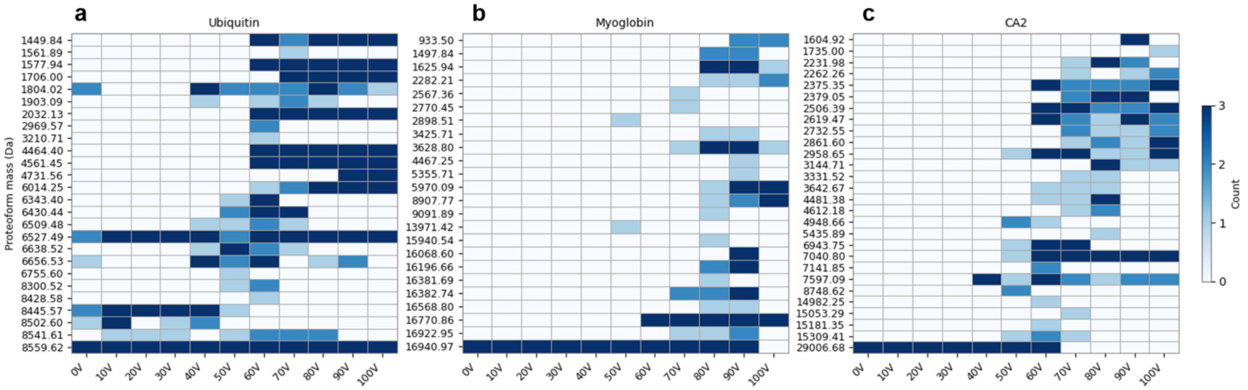

Fig. S5: ISF proteoforms identified at different ISF voltage settings. Proteoforms were ranked in the increasing order of their molecular masses. The color of each cell represents the number of technical MS replicates in which the proteoform was identified. (a) Ubiquitin, (b) myoglobin, and (c) CA2.

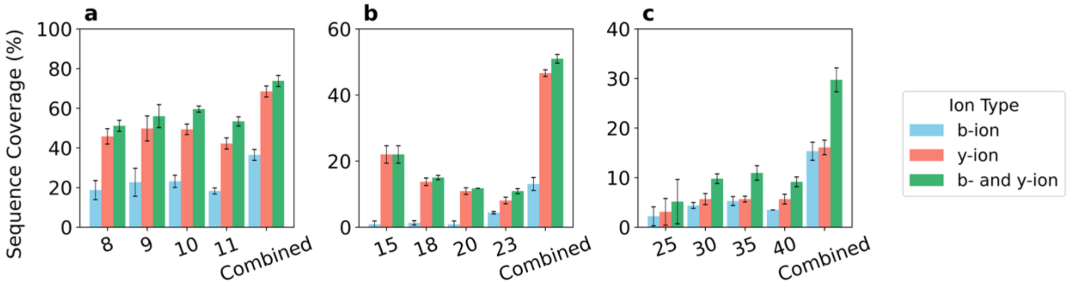

Fig. S6: Comparison of proteoform sequence coverage using representative PrSMs of single charge states versus combining representative PrSMs of multiple charge states. Sequence coverage obtained by representative PrSMs of the reference proteoform from triplicate MS runs at an ISF energy of 0 V are shown. (a) Single charge states 8, 9, 10, 11 are compared with combining multiple charge states 8 - 11 for ubiquitin. (b) Single charge states 15, 18, 20, 23 are compared with combining multiple charge states 15 - 23 for myoglobin. (c) Single charge states 26, 30, 35, 40 are compared with combining multiple charge states 25 - 40 for CA2.

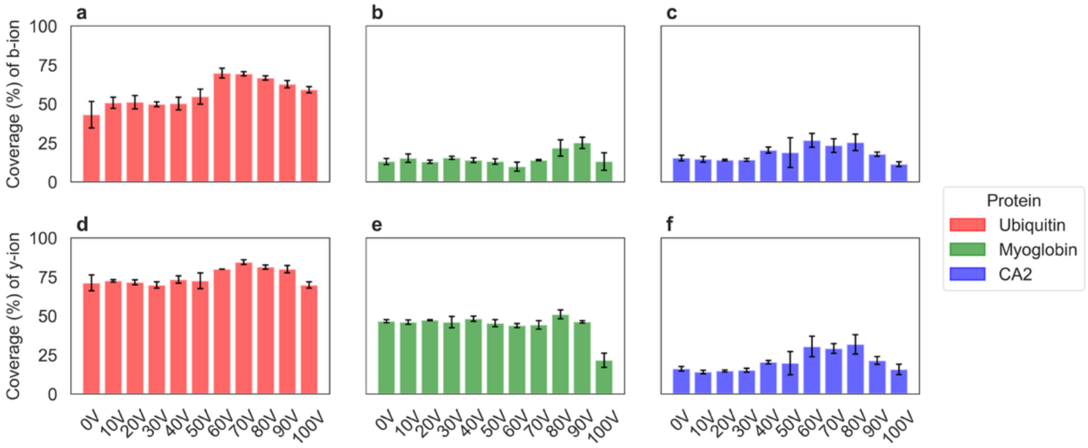

Fig. S7: Comparison of proteoform sequence coverage from b-ions and y-ions across different ISF voltage settings. B-ion coverage for (a) ubiquitin, (b) myoglobin, and (c) CA2, and y-ion coverage for (d) ubiquitin, (e) myoglobin, and (f) CA2. Error bars indicate standard deviations across triplicates.

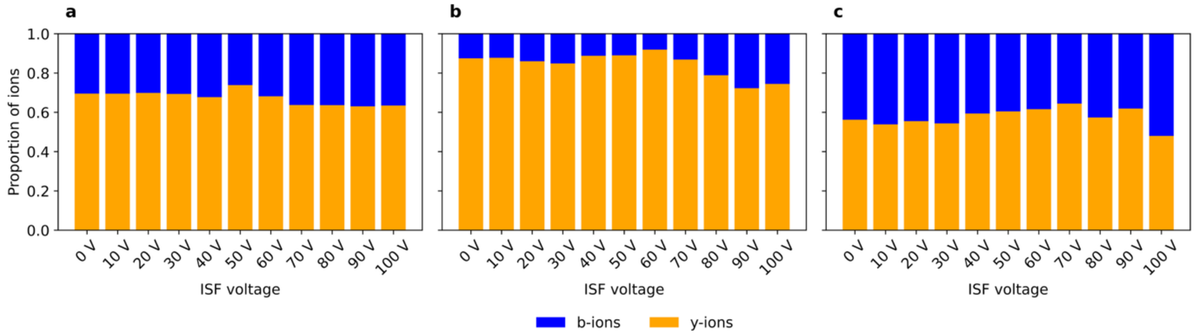

Fig. S8: Percentages of b- and y-ions in proteoforms identified by top-down MS with various ISF energy settings. Percentages of b- and y-ions in proteoforms identified from replicate 1 MS runs across all ISF voltages for (a) Ubiquitin, (b) myoglobin, and (c) CA2.

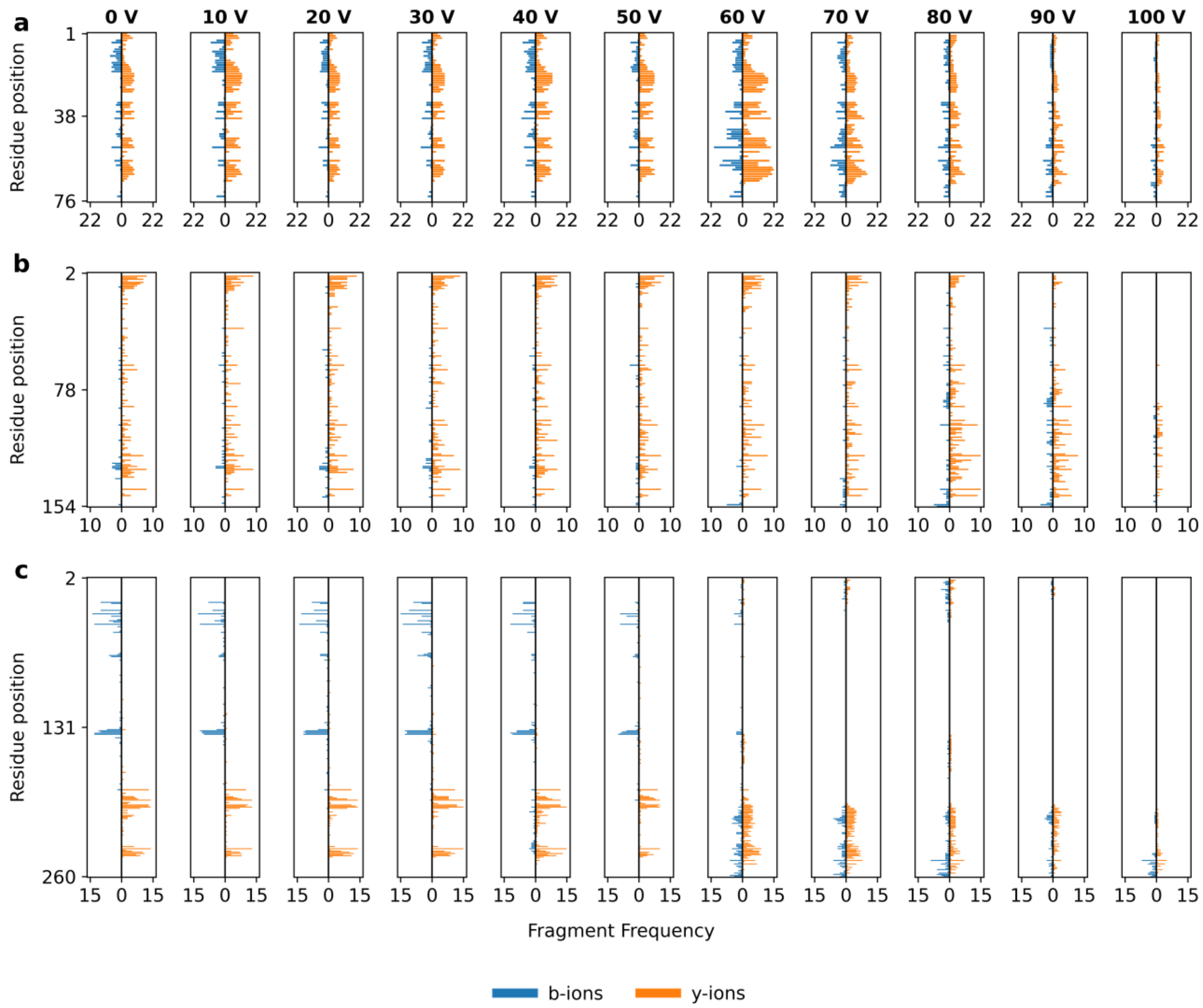

Fig. S9: Distributions of the cleavage sites of fragment ions of proteoforms identified by top-down MS with various ISF energy settings. Distributions of b- and y-ion cleavage sites along the protein sequences of (a) ubiquitin, (b) myoglobin, and (c) CA2 across all ISF voltages for replicate 1 MS runs.

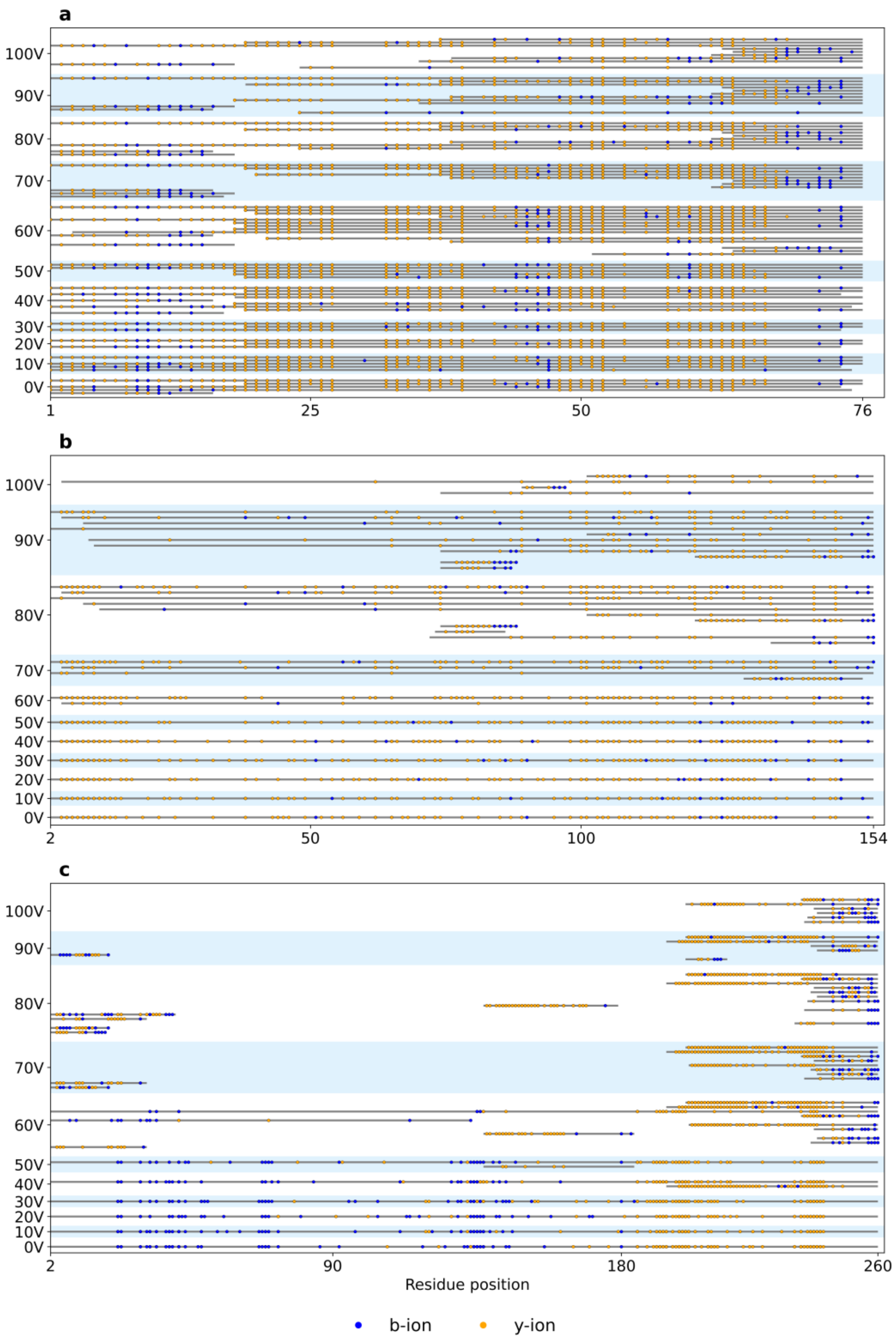

Fig. S10. Fragment ion sequence coverage obtained using proteoforms identified by top-down MS with various ISF energy settings. Cleavage sites of b- and y-ions for each proteoform identified in replicate 1 MS runs across all ISF voltages for (a) ubiquitin, (b) myoglobin, and (c) CA2. Each line represents a proteoform and each dot represents the cleavage site of a fragment ion.

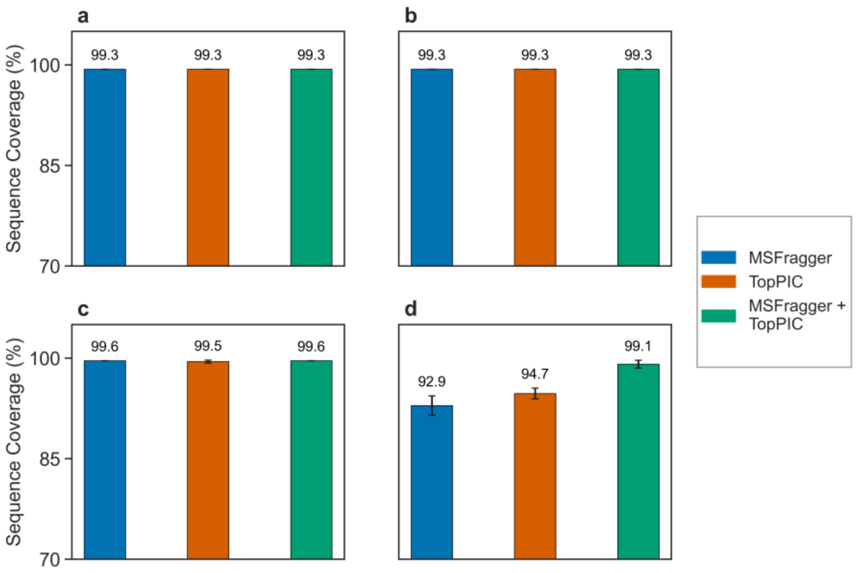

Fig. S11: Comparison of sequence coverage for myoglobin and CA2 in middle-down MS using five enzymes. Sequence coverage comparison among the MSFragger-only method, the TopPIC-only method, and the combined method using data from all five enzymes: (a) myoglobin with CID, (c) myoglobin with HCD, (b) CA2 with CID, and (d) CA2 with HCD.

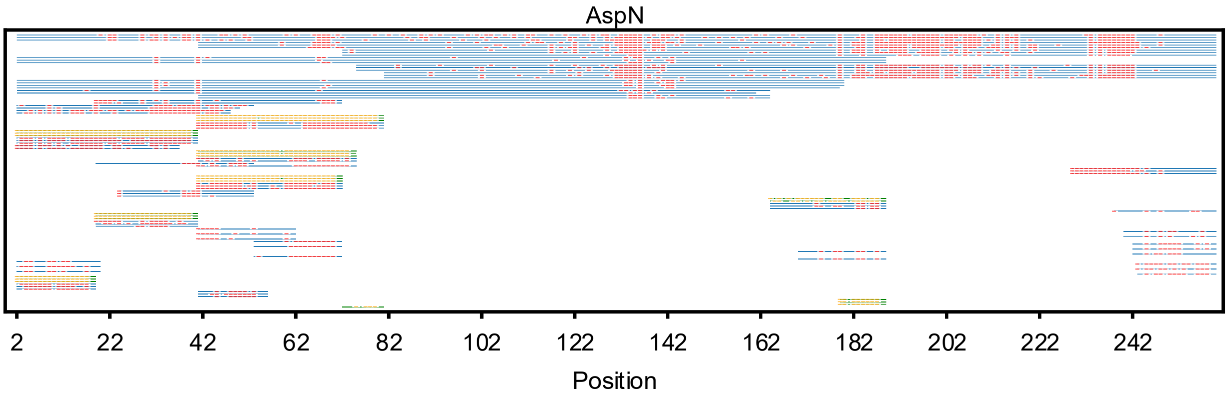

Fig. S12: Peptides and proteoforms of CA2 identified using MSFragger and TopPIC by middle-down MS with AspN digestion. Identifications are from the first replicate of the CID runs. Each blue line with red ticks represents a peptide/proteoform identified by TopPIC, and each green line with orange ticks represents a peptide/proteoform identified by MSFragger. Red and orange ticks indicate the cleavage sites of matched fragment masses.

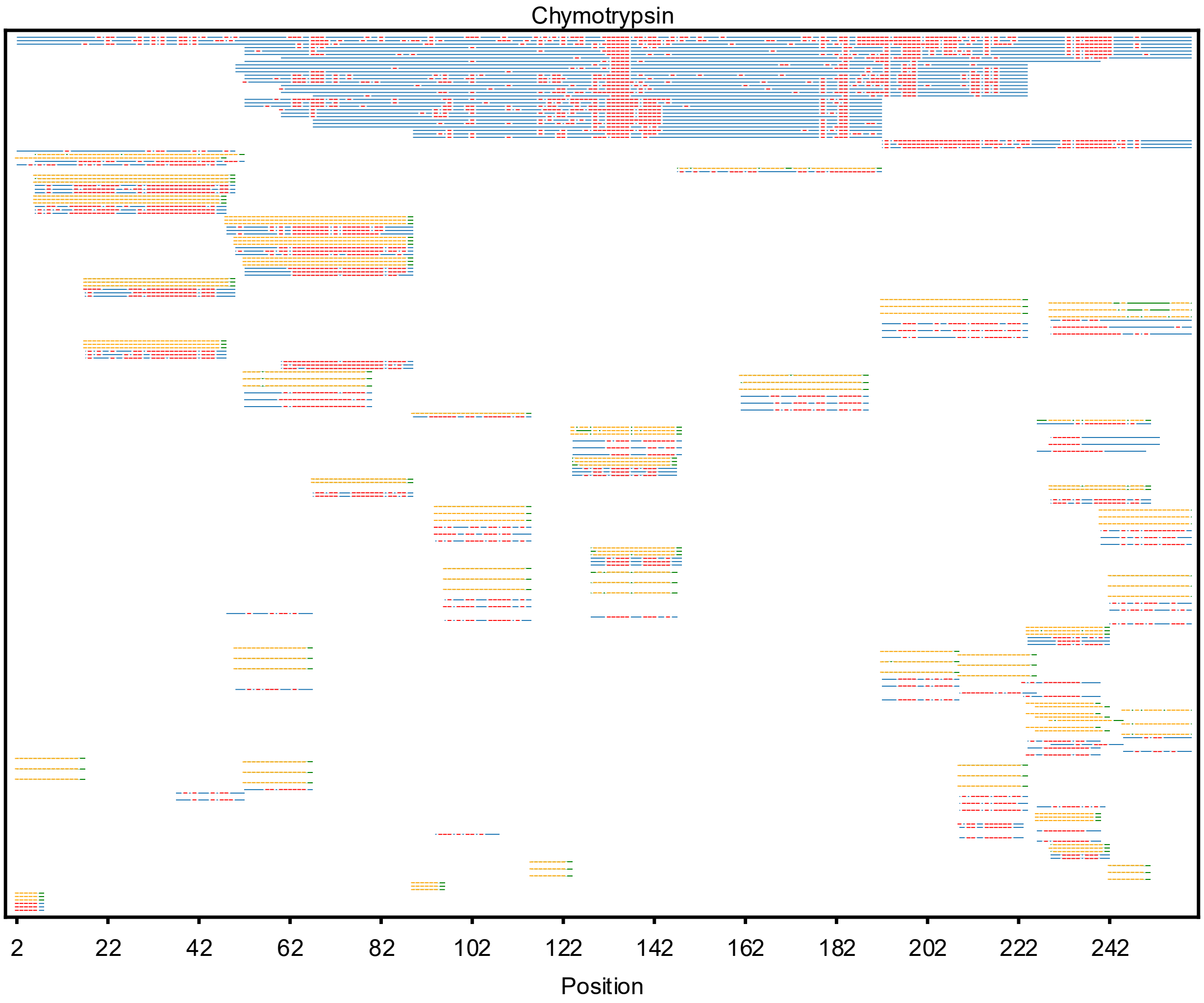

Fig. S13: Peptides and proteoforms of CA2 identified using MSFragger and TopPIC by middle-down MS with chymotrypsin digestion. Identifications are from the first replicate of the CID runs. Each blue line with red ticks represents a peptide/proteoform identified by TopPIC, and each green line with orange ticks represents a peptide/proteoform identified by MSFragger. Red and orange ticks indicate the cleavage sites of matched fragment masses.

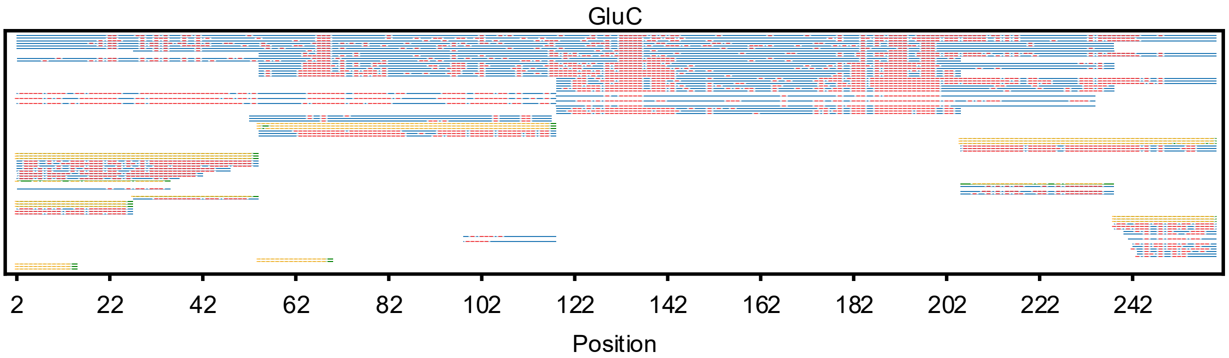

Fig. S14: Peptides and proteoforms of CA2 identified using MSFragger and TopPIC by middle-down MS with GluC digestion. Identifications are from the first replicate of the CID runs. Each blue line with red ticks represents a peptide/proteoform identified by TopPIC, and each green line with orange ticks represents a peptide/proteoform identified by MSFragger. Red and orange ticks indicate the cleavage sites of matched fragment masses.

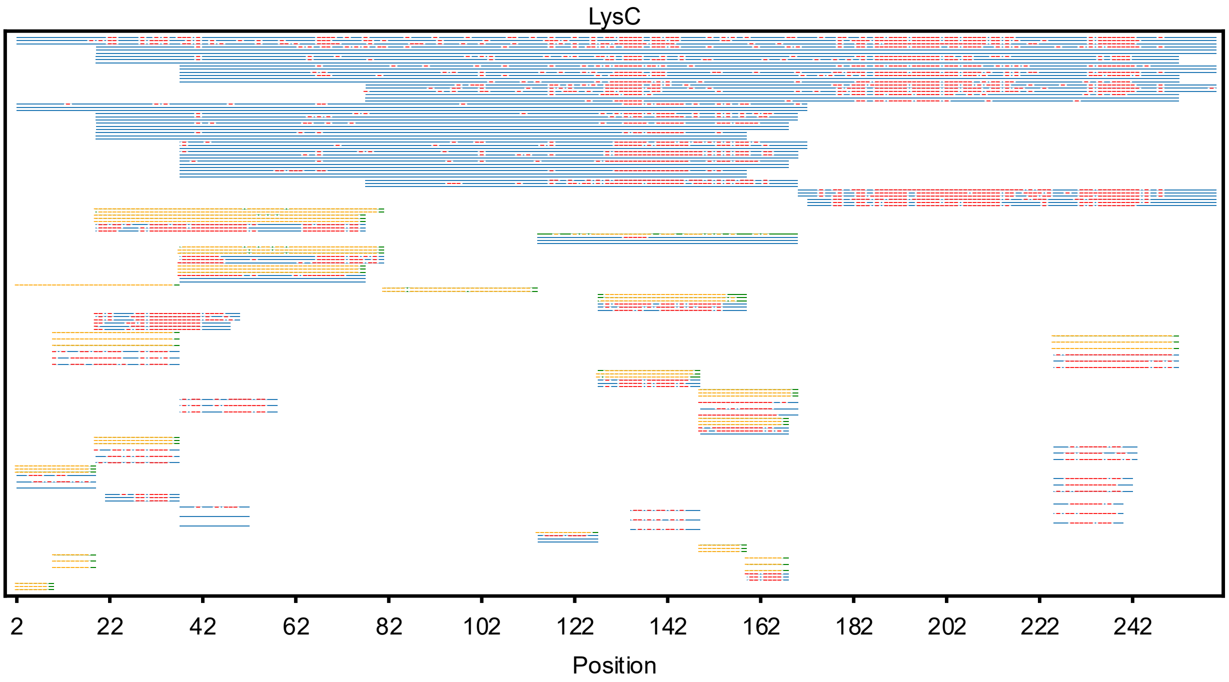

Fig. S15: Peptides and proteoforms of CA2 identified using MSFragger and TopPIC by middle-down MS with LysC digestion. Identifications are from the first replicate of the CID runs. Each blue line with red ticks represents a peptide/proteoform identified by TopPIC, and each green line with orange ticks represents a peptide/proteoform identified by MSFragger. Red and orange ticks indicate the cleavage sites of matched fragment masses.

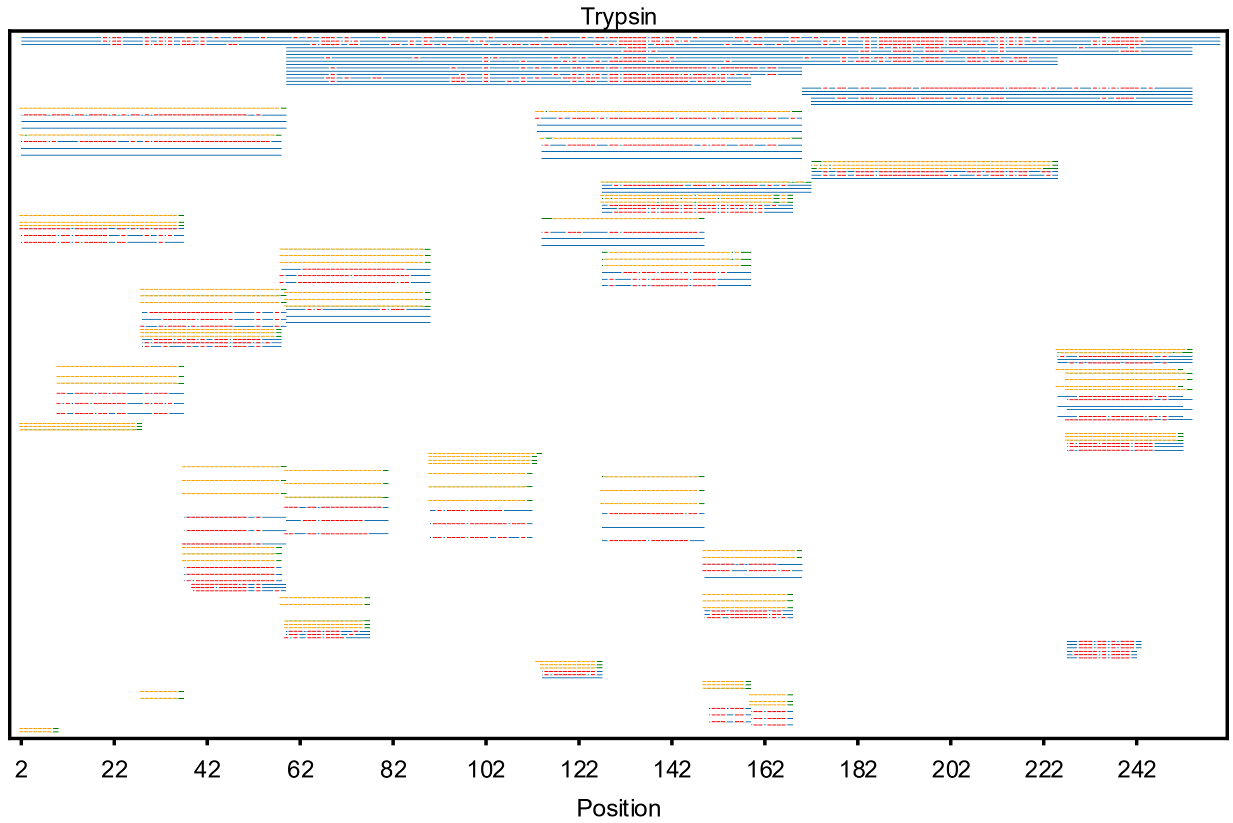

Fig. S16: Peptides and proteoforms of CA2 identified using MSFragger and TopPIC by middle-down MS with trypsin digestion. Identifications are from the first replicate of the CID runs. Each blue line with red ticks represents a peptide/proteoform identified by TopPIC, and each green line with orange ticks represents a peptide/proteoform identified by MSFragger. Red and orange ticks indicate the cleavage sites of matched fragment masses.
